# Atenolol Induced HDL-C Change in the Pharmacogenomic Evaluation of Antihypertensive Responses (PEAR) Study

**DOI:** 10.1371/journal.pone.0076984

**Published:** 2013-10-07

**Authors:** Caitrin W. McDonough, Nancy K. Gillis, Abdullah Alsultan, Shin-Wen Chang, Marina Kawaguchi-Suzuki, Jason E. Lang, Mohamed Hossam A. Shahin, Thomas W. Buford, Nihal M. El Rouby, Ana C.C. Sá, Taimour Y. Langaee, John G. Gums, Arlene B. Chapman, Rhonda M. Cooper-DeHoff, Stephen T. Turner, Yan Gong, Julie A. Johnson

**Affiliations:** 1 Department of Pharmacotherapy and Translational Research and Center for Pharmacogenomics, University of Florida, College of Pharmacy, Gainesville, Florida, United States of America; 2 Division of Pulmonary and Sleep Medicine and Center for Pharmacogenomics and Translational Research, Nemours Children’s Hospital, Orlando, Florida, United States of America; 3 Department of Aging and Geriatric Research, University of Florida, College of Medicine, Gainesville, Florida, United States of America; 4 Department of Community Health and Family Medicine, University of Florida, College of Medicine, Gainesville, Florida, United States of America; 5 The Renal Division, Department of Medicine, Emory University, Atlanta, Georgia, United States of America; 6 Division of Cardiovascular Medicine, Department of Medicine, University of Florida, Gainesville, Florida, United States of America; 7 Division of Nephrology and Hypertension, Mayo Clinic, Rochester, Minnesota, United States of America; Children's National Medical Center, Washington, United States of America

## Abstract

We sought to identify novel pharmacogenomic markers for HDL-C response to atenolol in participants with mild to moderate hypertension. We genotyped 768 hypertensive participants from the Pharmacogenomic Evaluation of Antihypertensive Responses (PEAR) study on the Illumina HumanCVD Beadchip. During PEAR, participants were randomized to receive atenolol or hydrochlorothiazide. Blood pressure and cholesterol levels were evaluated at baseline and after treatment. This study focused on participants treated with atenolol monotherapy. Association with atenolol induced HDL-C change was evaluated in 232 whites and 152 African Americans using linear regression. No SNPs achieved a Bonferroni corrected *P*-value. However, we identified 13 regions with consistent association across whites and African Americans. The most interesting of these regions were seven with prior associations with HDL-C, other metabolic traits, or functional implications in the lipid pathway: *GALNT2*, *FTO*, *ABCB1, LRP5*, *STARD3NL*, *ESR1*, and *LIPC*. Examples are rs2144300 in *GALNT2* in whites (*P*=2.29x10^-4^, β=-1.85 mg/dL) and rs12595985 in *FTO* in African Americans (*P*=2.90x10^-4^, β=4.52 mg/dL), both with consistent regional association (*P*<0.05) in the other race group. Additionally, baseline *GALNT2* expression differed by rs2144300 genotype in whites (*P*=0.0279). In conclusion, we identified multiple gene regions associated with atenolol induced HDL-C change that were consistent across race groups, several with functional implications or prior associations with HDL-C.

## Introduction

Hypertension is a major risk factor for cardiovascular disease (CVD), which is the leading cause of morbidity and mortality in the United States [1]. Over 76 million Americans have hypertension, of which an estimated 71% are taking antihypertensive medications [1]. Thiazide diuretics, β-blockers, angiotensin converting enzyme inhibitors, angiotensin receptor blockers, and calcium channel blockers are all acceptable first-line treatments for hypertension [2]. However, there has been debate about the first-line role of β-blockers and diuretics due to their adverse metabolic effects on glucose, insulin, and lipid levels [3]. For example, β-blockers have been shown to significantly decrease high-density lipoprotein cholesterol (HDL-C) levels, but the underlying mechanism is unclear [4]. These metabolic effects, including HDL-C reduction, are also strong contributors to the risk of coronary heart disease and stroke [1].

Data from four large, prospective studies, including The Framingham Heart Study, suggest that every 1 mg/dL (0.026 mmol/L) decrease in HDL-C is associated with a 2-3% increase in risk for coronary heart disease [5]. Additionally, post hoc analysis of the Treating to New Targets (TNT) trial demonstrated that low levels of HDL-C were predictive of higher rates of CVD, even at very low levels of low-density lipoprotein cholesterol (LDL-C) (< 70 mg/dL) [6] and low HDL-C levels were also predictive of major cardiovascular events in patients treated with statins [7]. However, randomized controlled trials designed to determine if increases in HDL-C prevent coronary heart disease and major cardiovascular events demonstrate inconsistent results [8,9]. Additionally, it remains unclear if decreases in HDL-C in response to β-blocker therapy are harmful in a similar way as low HDL-C observed in epidemiological studies.

Clear data exist regarding the influence of genetics on HDL-C levels. In a study of the sex specific genetic architecture of many quantitative traits, the heritability of HDL-C was estimated at 52% in males and 70% in females [10]. HDL-C is a sexually dimorphic trait, with higher HDL-C levels typically observed in females [10]. There have been multiple genome-wide association studies (GWAS) investigating the effect of genetic variants on blood lipid levels. In a large-scale meta-analysis including many of these GWAS, 45 loci were found to influence HDL-C [11]. When examining the entire set of single nucleotide polymorphisms (SNPs) associated with HDL-C, 12.1% of the total variance in HDL-C was explained, and ~25-30% of the genetic variance was explained [11].

While there have been many studies investigating genetic influences on blood lipid levels, including HDL-C [11], the genetics of HDL-C response to β-blockers remains unexplored. In order to evaluate potential genetic effects, we assessed SNPs across the genome from the Illumina HumanCVD chip [12] for association with atenolol induced HDL-C change in the Pharmacogenomic Evaluation of Antihypertensive Responses (PEAR) study [13].

## Methods

### Ethics Statement

All study participants provided written informed consent and the study protocol was approved by the Institutional Review Boards at all participating clinical trial study sites: University of Florida, Mayo Clinic, and Emory University. The study was conducted according to the principles expressed in the Declaration of Helsinki.

### Study Design and Participants

The PEAR study was a prospective, multicenter, randomized, open-label study (clinicaltrials.gov NCT00246519). The design of the PEAR study has been described previously [13]. Briefly, PEAR included 768 participants with mild to moderate hypertension between the ages of 17 to 65, without other comorbidities including diabetes. Participants were included if they had an average home diastolic blood pressure (DBP) between 86 and 110 mmHg and office DBP between 91 and 110 mmHg at the end of an average 4 week washout period. After washout, study participants were randomized to receive atenolol 50 mg or hydrochlorothiazide (HCTZ) 12.5 mg daily. If BP remained greater than 120/70 mmHg after three weeks of treatment, doses were titrated to atenolol 100 mg or HCTZ 25 mg daily, and treatment was continued for an additional 6 weeks. The other agent was then added based on BP > 120/70 mmHg, with similar dose titration for 6-9 weeks of combination treatment. During atenolol monotherapy, >82% of whites and >88% of African Americans underwent the dose titration step. In addition to blood pressure measures, laboratory measures including HDL-C were acquired at baseline, after monotherapy, and after combination therapy [13].

### Determination of Blood Lipid Concentration

Fasting serum levels of lipids (including HDL-C) were determined using a Hitachi 911 Chemistry Analyzer (Roche Diagnostics, Indianapolis, IN). All laboratory parameters were measured at a central laboratory at the Mayo Clinic. All samples were tested in duplicate, with the means of the duplicate samples reported.

### SNP Genotyping and Quality Control

DNA from study participants was genotyped on the Illumina HumanCVD Beadchip (Illumina, San Diego, CA). This is a gene-centric array containing ~50,000 SNPs in ~2,100 genes involved in cardiovascular, inflammatory, and metabolic processes [12]. Genotyping was performed on Illumina’s iScan System using the Infinium II Assay (Illumina, San Diego, CA). Genotypes were called using GenomeStudio Software version 2011.1 and the Genotyping Module version 1.9 calling algorithm (Illumina, San Diego, CA). Samples were excluded if genotype call rates were below 90% and SNPs were excluded if genotype call rates were below 90%. Eighty-one blind duplicates were included in genotyping and had a concordance rate of 99.992%. Gender was confirmed from X chromosome genotype data, and those who were discordant were excluded (*n*=1). Cryptic relatedness was estimated by pairwise identity-by-descent (IBD) analysis implemented using PLINK (http://pngu.mgh.harvard.edu/purcell/plink/) [14]. One pair of monozygotic twins was identified in this analysis and one twin was removed (*n*=1). Five pairs of samples were identified as first-degree relatives; these individuals were kept for the analyses. Heterozygosity was assessed using PLINK, by estimating the inbreeding coefficient, F. One sample had F values > 4 negative standard deviations from the mean and was excluded. The final genetic dataset consisted of 765 samples. This analysis focused on the 232 whites and 152 African-Americans randomized to the atenolol monotherapy arm.

### Statistical Analysis

To address the issue of population substructure and admixture in our racially and ethnically diverse population, a Principal Component Analysis (PCA) was performed in all samples on a linkage disequilibrium (LD) pruned dataset using the EIGENSTRAT method [15]. Race/ethnic groups were confirmed with PCA clustering results, and participants who identified with a race/ethnic group other than white or African American were collapsed into the group they clustered nearest. The top principal components (PCs 1-2) that provided the best separation of ancestry clusters were selected to be included as covariates for analysis.

Before analysis, all SNPs with a minor allele frequency (MAF) < 3% were removed from the data set due to limited power. This was performed separately for each race group, leaving 33,785 SNPs for analysis in the white race group, and 39,718 SNPs for analysis in the African Americans. SNPs were tested for departure from Hardy-Weinberg Equilibrium (HWE) in each race group using an exact test. Linear regression was used to model SNP effects on HDL-C response to atenolol monotherapy using PLINK (http://pngu.mgh.harvard.edu/purcell/plink/) [14]. The primary analysis was based on the additive genetic model. Analyses were adjusted for age, sex, baseline HDL-C, and principal components for ancestry. Additional covariates were investigated (BMI, exercise, smoking status, alcohol use, baseline LDL, baseline glucose, lipid lowering medications, and fish oil supplementation), but none were shown to be predictive of HDL-C response in our population, and therefore, were not included as covariates.

Statistical significance was defined as a Bonferroni corrected *P*-value for the number of independent SNPs (*P*<2.4x10^-6^ in whites and *P*<1.9x10^-6^ in African Americans) [16]. Since no SNPs achieved these *P*-values, top hits of interest were identified as regions showing evidence of association in both race groups. The screening threshold was set at *P*<1x10^-3^ in either race group. SNPs meeting the screening *P*-value were examined in the other race group for evidence of association. Next, the gene region (gene ± 10kb) was also examined for evidence of association, as patterns of LD often differ between race groups and different tag SNPs could be observed in each race group. Regional SNPs were not required to have effects in the same direction, as due to differences in racial LD and allele frequency, the different alleles could be tagging the same unknown causal variant. The significance threshold for validation within the gene region in the second race group was *P*<0.005, or *P*<0.05 if there was evidence from previous HDL-C studies or the encoded protein plays a clear role in HDL-C/lipid trafficking/metabolism. Adjusted mean change by genotype was calculated for the top hits using the general linear models procedure in SAS version 9.2 (SAS Institute Inc., Cary, North Carolina, USA). Additionally, top hits with low minor allele frequencies (<0.05) were investigated to determine if single individuals were strongly contributing to the observed association. One white participant was identified with an extreme HDL-C response, standardized residual value from linear regression including non-genetic covariates outside 4 standard deviations, and was removed in a sensitivity analysis. Similarly, for rs12595985 in *FTO*, sensitivity analyses were also run using a dominant genetic model, collapsing the AA homozygote with the AC heterozygotes, and using an additive genetic model after removing the single African American AA homozygote.

FastSNP and SNPNexus were used to predict the potential functional consequences of the top hits [17,18]. Results of association were displayed using Haploview, WGAViewer, and LocusZoom [19-21].

### Gene Expression Analysis

Gene expression analysis was performed for the top genes that were 1) previously associated with HDL-C or lipid trafficking/metabolism, 2) expressed in lymphocytes, and 3) had RNA available for at least 10 minor allele homozygotes and/or 10 minor allele carriers. Three genes met these criteria: *GALNT2*, *FTO*, and *LRP5*; however, *LRP5* was expressed at extremely low levels in lymphocytes and was not tested further. Expression of *GALNT2* was measured in 34 PEAR whites (11 AA, 12 GA, and 11 GG at rs2144300) and expression of *FTO* was measured in 35 PEAR African Americans (11 AC, and 24 CC at rs12595985). RNA was isolated from whole blood using the PAXgene Blood RNA Kit IVD (Qiagen, Valenica, CA, USA), before atenolol treatment, and converted to cDNA. Gene expression was measured by quantitative real-time RT-PCR using Taqman Gene Expression Assays and the Taqman 7900HT Real Time PCR System (Applied Biosystems, Foster City, CA, USA). Expression levels were normalized to the reference gene β-2-microglobulin. Relative gene expression was calculated using the 2^-ΔCt^ method [22]. Expression levels between genotype groups at baseline (before atenolol treatment) were compared using a trend test (*GALNT2*) or an unpaired *t*-test (*FTO*). The significance threshold was set at *P*<0.05.

## Results

The results presented are for PEAR study participants on atenolol monotherapy (i.e. the atenolol arm). Baseline characteristics and demographics are shown in Table **1**. Among a total of 384 study participants in the atenolol group, 58.6% were white and 38.3% were African American. Participants were overweight (average BMI = 30.8 kg/m^2^), and 12.8% were current smokers. The average baseline HDL-C was 49.7 mg/dL.

**Table 1:** Baseline characteristics and demographics of PEAR study participants treated with atenolol monotherapy.

Characteristic	Atenolol (n=384)
Age (years)	48.6 ± 9.2
Female, n (%)	215 (56.0)
BMI (kg/m^2^)	30.8 ± 5.9
Waist circumference (cm)	97.6 ± 13.1
Race/ethnicity	
African American, n (%)	147 (38.3)
White, n (%)	225 (58.6)
Other, n (%)	12 (3.1)
Genetic Race/ethnicity	
African American, n (%)	152 (39.6)
White, n (%)	232 (60.4)
Current smoker, n (%)	49 (12.8)
Clinic Blood Pressure (mm Hg)	
Systolic	151.2 ± 12.3
Diastolic	98.3 ± 6.2
Fasting Laboratory Measures	
Glucose (mg/dL)	91.1 ± 11.9
Triglycerides (mg/dL)	122.4 ± 84.2
LDL-C (mg/dL)	119.1 ± 33.4
HDL-C (mg/dL)	49.7 ± 14.8

Values are presented as mean ± standard deviation, unless otherwise noted. BMI: Body Mass Index, LDL-C: low-density lipoprotein cholesterol, HDL-C: high-density lipoprotein cholesterol

The Manhattan plots and corresponding Q-Q plots for HDL-C response to atenolol are shown in Figure **S1** and Figure **S2**, respectively. No SNPs achieved a Bonferroni correct *P*-value. Overall, 25 SNPs met the screening *P*-value (*P*<1x10^-3^) in whites and 42 SNPs met the screening *P*-value in the African Americans; however, no SNP replicated in the other race group, in the same direction. After examining the identified gene regions in the other race for evidence of consistent association, thirteen regions met our threshold for association (*P*<0.005 or *P*<0.05 with prior HDL-C or lipid association, Table **2**). The allele frequencies, genotype counts, and Hardy-Weinberg equilibrium *P*-values for these SNPs are shown in Table **S1**. All SNPs identified in these gene regions were in Hardy-Weinberg Equilibrium (Hardy-Weinberg *P*-value > 0.01).

**Table 2:** Top gene regions associated with atenolol induced change in HDL-C^a^.

Locus				Initial Signal					Regional Validation Signal				
**Chr**	Position (Mb)	Gene	Prior HDL or Lipid Association [Ref]	Lead SNP	MA	MAF	Effect (mg/dl)	*P*-value	Lead SNP	MA	MAF	Effect (mg/dl)	*P*-value
				Whites					African Americans				
**1**	185.2	PLA2G4A		rs10157410	C	0.101	3.46	6.66E-05	rs4648287	G	0.049	3.21	0.0040
**1**	228.4	GALNT2	Yes [11]	rs2144300	G	0.422	-1.85	2.29E-04	rs2144297	A	0.345	-1.92	0.0233
**7**	38.2	STARD3NL	Yes [28]	rs10240718	A	0.078	3.23	3.98E-04	rs7795499	A	0.329	1.76	0.0350
**11**	68.0	LRP5	Yes [27]	rs3736228	A	0.136	2.64	5.07E-04	rs4988331	A	0.168	2.12	0.0281
**13**	77.4	EDNRB		rs3818416	A	0.241	2.21	2.97E-04	rs3818416	A	0.329	-2.24	0.0028
**15**	56.6	LIPC	Yes [11]	rs9652472	G	0.041	-4.40	6.02E-04	rs10518978	A	0.123	-3.01	0.0101
**16**	65.5	CDH16		rs3743725	A	0.030	5.23	7.51E-04	rs13336470	A	0.322	2.63	0.0041
				African Americans					Whites				
**4**	57.5	REST		rs6847086	A	0.461	2.67	7.14E-04	rs4109037	A	0.136	2.33	0.0025
**6**	152.5	ESR1	Yes [29]	rs3020384	C	0.487	2.65	7.63E-04	rs12199198	C	0.067	-2.07	0.0372
**7**	87.1	ABCB1	Yes [26]	rs3213619	G	0.072	5.22	2.74E-04	rs10267099	G	0.220	1.62	0.0059
**8**	10.1	MSRA		rs2975721	T	0.385	2.74	4.69E-04	rs6601419	A	0.338	1.66	0.0029
**13**	94.5	ABCC4		rs7319001	A	0.095	4.92	5.39E-04	rs1189470	C	0.154	2.12	0.0036
**16**	52.4	FTO	Yes [25]	rs12595985	A	0.109	4.52	2.90E-04	rs9940629	A	0.448	1.56	0.0041

Chr: Chromosome; Mb: Megabase; MA: Minor Allele; MAF: Minor Allele Frequency

^a^Regional validation SNPs were not required to have effects in the same direction, as due to differences in racial LD and allele frequency, the different alleles could be tagging the same unknown causal variant.

In whites, the strongest association was rs10157410 in *PLA2G4A* (*P*=6.66x10^-5^, beta=3.46, Table **2**), with consistent regional association at rs4648287 in the African Americans (*P*=0.0040, beta=3.21). *PLA2G4A* is located at chromosome 1q31.1 and encodes the cytosolic phospholipase A2. In African Americans, the strongest association was seen at rs3213619 on chromosome 7q21.12, with a *P*-value of 2.74x10^-4^ and a beta of 5.22 (Table **2**). rs3213619 lies in *ABCB1*, ATP-binding cassette sub-family B member 1, and consistent regional association was also observed at rs10267099 in whites (*P*=0.0059, beta=1.62).

**Figure 1:**
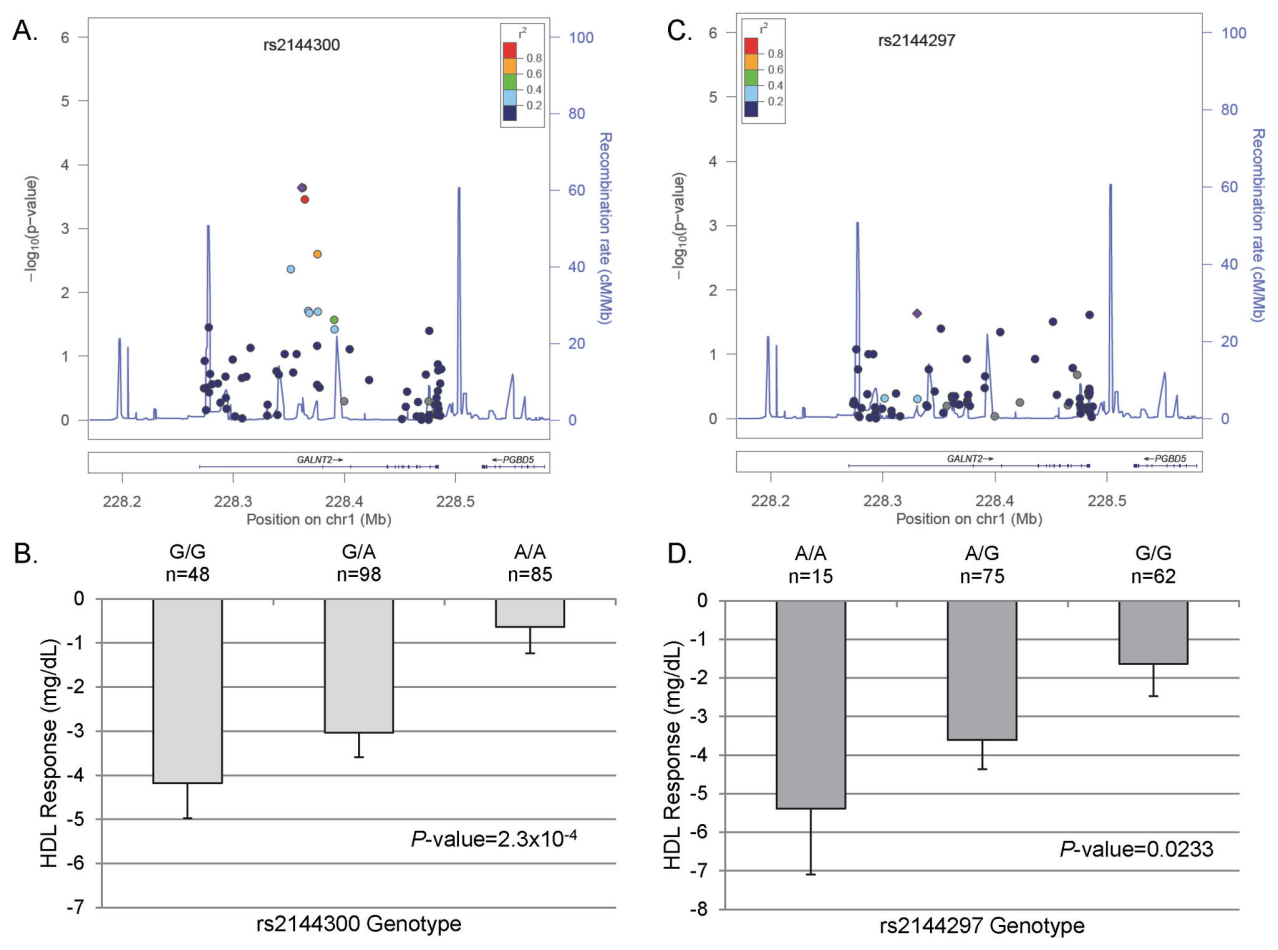
*GALNT2* Regional Plots and adjusted HDL-C response by genotype. (A) Regional Plot in PEAR whites. (B) HDL-C response in PEAR whites by rs2144300 genotype. (C) Regional Plot in PEAR African Americans. (D) HDL-C response in PEAR African Americans by rs2144297 genotype. Error bars represent standard error.

Two other regions are of particular note. First, *GALNT2*, polypeptide N-acetylgalactosaminyltransferase 2, which has been previously associated in GWAS studies with circulating HDL-C levels [11], was associated with atenolol induced HDL-C change. In whites the G allele of rs2144300, one of the same SNPs identified from GWAS studies [23], was associated with an atenolol induced decrease in HDL-C (*P*=2.29x10^-4^, Table **2**, Figure **1A**), with a mean HDL-C change of -4.18 mg/dL, -3.03 mg/dL, and -0.63 mg/dL for the GG, GA, and AA genotypes, respectively (Figure **1B**). In African Americans, a signal was observed in *GALNT2* with the A allele of rs2144297 and a decrease in HDL-C in response to atenolol (*P*=0.0233, Table **2**, Figure **1C**). The mean change in HDL-C was -5.39 mg/dL, -3.61 mg/dL, and -1.63 mg/dL for the AA, AG, and GG genotypes, respectively (Figure **1D**).

**Figure 2:**
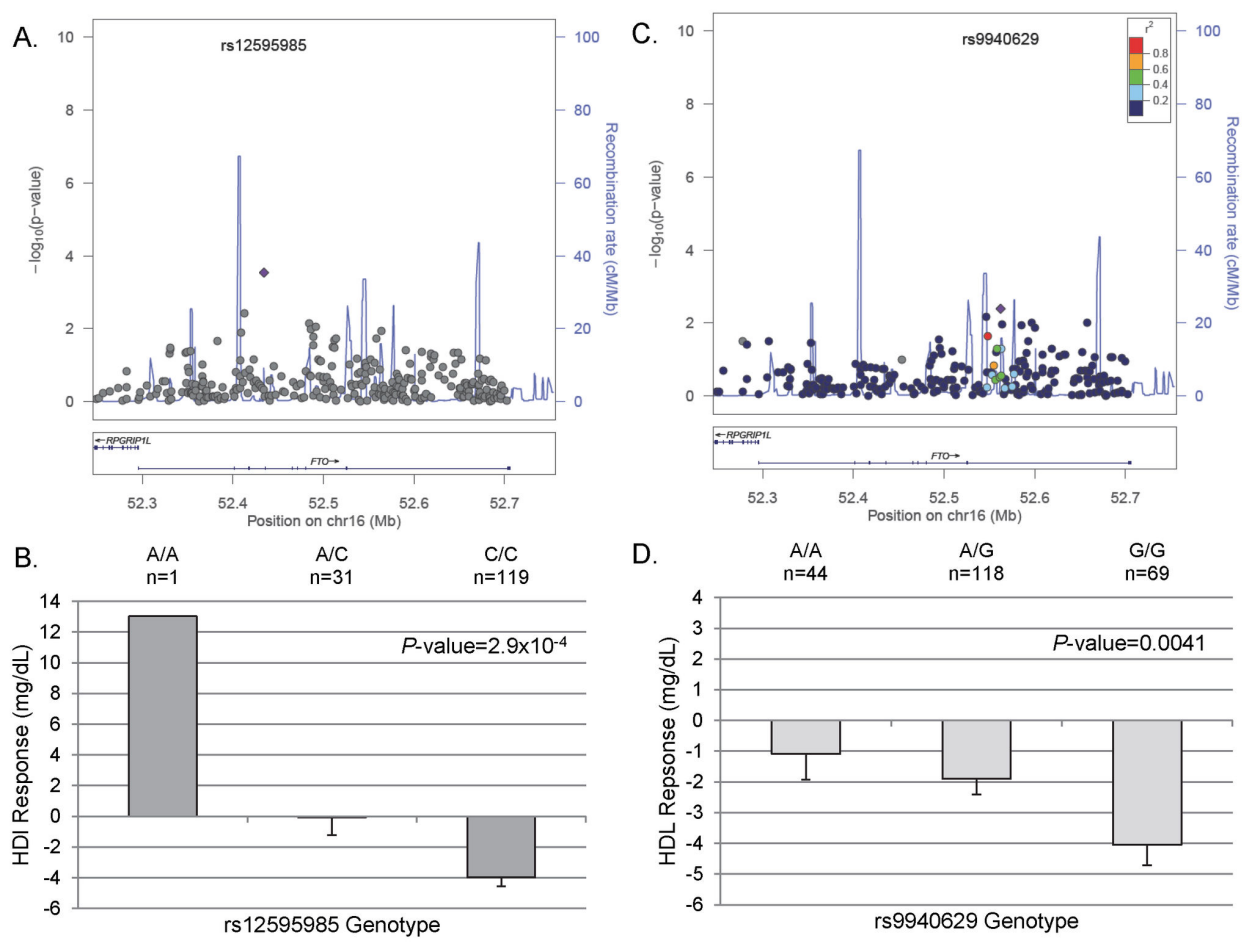
*FTO* Regional Plots and adjusted HDL-C response by genotype. (A) Regional Plot in PEAR African Americans. (B) HDL-C response in PEAR African Americans by rs12595985 genotype. (C) Regional Plot in PEAR whites. (D) HDL-C response in PEAR whites by rs9940629 genotype. Error bars represent standard error.

The second gene, *FTO*, the fat mass and obesity associated gene, has been previously associated with BMI, obesity and type 2 diabetes in GWAS [24], and was recently associated with HDL-C in a large, gene-centric analysis of the CVD array used herein [25]. In African Americans, the A allele of rs12595985 was associated with an increase in HDL-C in response to atenolol treatment, with an increase of 13.03 mg/dL in the AA homozygote, an average decrease of 0.07 mg/dL in AC heterozygotes, and an average decrease of 3.97 mg/dL in CC homozygotes (*P*=2.90x10^-4^, Table **2**, Figures **2A** and **2B**). The observed signal in whites was with rs9940629, with the G allele associated with a greater reduction in HDL-C after atenolol therapy (*P*=0.0041, Table **2**, Figure **2C**). The mean change in HDL-C was -1.09 mg/dL, -1.90 mg/dL, and -4.04 mg/dL for the AA, AG, and GG genotypes, respectively (Figure **2D**).

Top initial signals with low minor allele frequencies were further investigated to determine if single study participants with an extreme HDL-C response to atenolol were driving the observed signals. One white participant with an HDL-C change of -36.4 mg/dL was identified and the individual was removed in a sensitivity analysis (Table **S2**). All of the initial signals in whites remained except for *LIPC* rs9652472 (sensitivity *P*-value=0.3998). Additionally, sensitivity analyses were conducted in African Americans at rs12595985 in *FTO* (Table **S3**). The signal remained under both a dominant genetic model, collapsing the AA homozygote with the AC heterozygotes (sensitivity *P*-value=9.71x10^-4^) and an additive genetic model, after removing the single AA homozygote, (sensitivity *P*-value=3.18x10^-3^).

The putative functional implications of the lead SNPs from the initial signals in the 13 gene regions are shown in Table **S4**. rs3736228, in *LRP5* (low density lipoprotein receptor-related protein 5), is a non-synonymous SNP causing a conservative amino acid change of an alanine to valine at amino acid position 1330. Additionally, rs3736228 was predicted by FastSNP to affect splicing regulation. All of the other SNPs identified were located in intronic regions, or upstream or downstream of the nearest gene.

**Figure 3:**
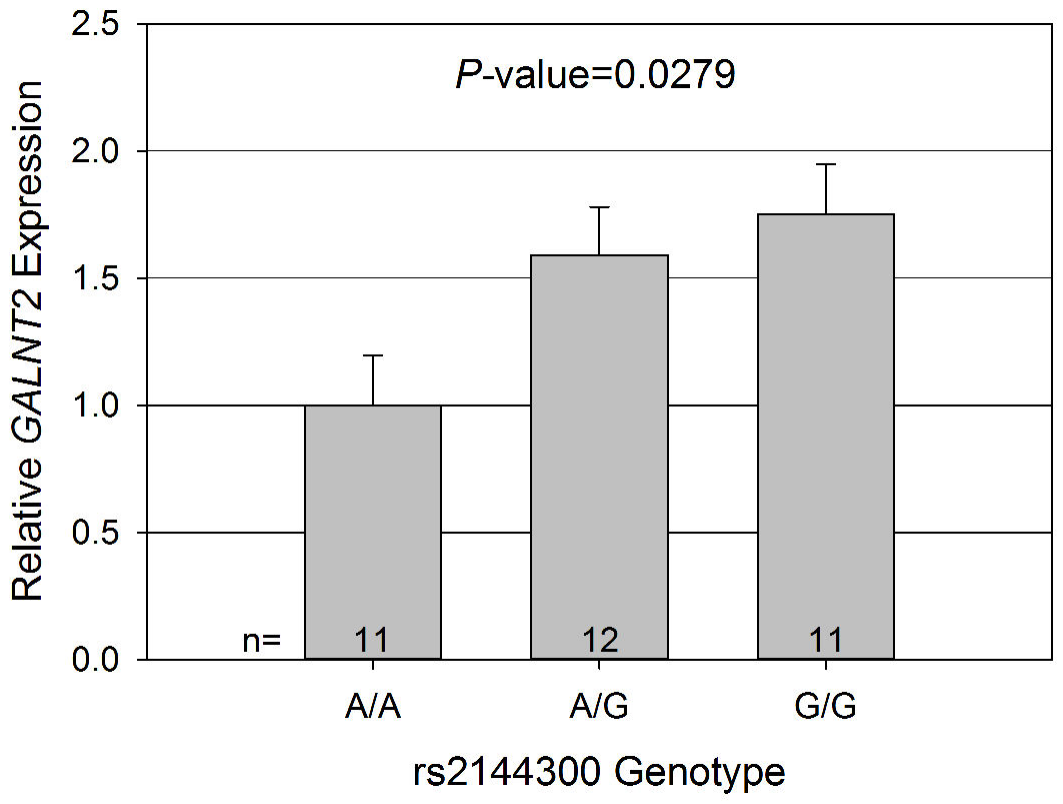
Plot of relative gene expression of *GALNT2* by rs2144300 genotype. Gene expression analysis performed in 34 PEAR whites, normalized to β-2-microglobulin. Error bars represent standard error.

In order to examine the possible function of two the top SNPs, we measured gene expression at baseline (before atenolol treatment) of *GALNT2* by rs2144300 genotype in 34 whites and of *FTO* by rs12595985 genotype in 35 African Americans. For *GALNT2*, study participants with one or two copies of the G allele had a 1.59 and 1.75 fold increase in relative expression at baseline, respectively, compared to AA homozygotes (*P*=0.0279, Figure **3**). There was no difference in baseline expression between genotype groups for *FTO* (data not shown).

## Discussion

We report, for the first time to our knowledge, a genome-spanning analysis using the HumanCVD Beadchip, which contains cardiovascular, metabolic and inflammatory candidate genes, to identify genetic variants associated with atenolol induced changes in HDL-C in hypertensive study participants. While no SNPs achieved a Bonferroni corrected *P*-value, using a staged analysis approach that required consistent regional association in white and African American participants, we identified 7 SNPs associated with HDL-C response to atenolol in whites that showed evidence of consistent regional association in African Americans. Additionally, 6 SNPs were found in African Americans with consistent regional association in whites. Most notable were seven gene regions with prior associations with HDL-C or lipid metabolism/trafficking: *GALNT2, FTO*, *ABCB1*, *LRP5*, *STARD3NL*, *ESR1*, and *LIPC* [11,25-29].

*GALNT2*, located at 1q42.13, is a member of the GalNAc-transferase family, which transfers N-acetyl galactosamine to the hydroxyl group of a serine or threonine residue in the first step of O-linked oligosaccharide biosynthesis [30]. Common variants in *GALNT2* have previously been associated with both HDL-C and triglyceride levels in numerous GWAS analyses, and subsequently validated [11,23]. Additionally, altered expression of *Galnt2* in mouse models, both knockdown and over expression, has been inversely correlated with altered HDL-C levels [11]. This is similar to what we observed; where those individuals with higher gene expression at baseline (G carriers at rs2144300), had greater decreases in HDL-C in response to atenolol, resulting in lower HDL-C levels. These prior data, the association of common SNPs in this gene with HDL-C response to atenolol in whites and African Americans, and the difference in *GALNT2* expression by rs2144300 genotype in whites suggest *GALNT2* may be an important mediator of the observed atenolol associated HDL-C response.

*FTO*, located at 16q12.2, is thought to be involved in the regulation of food intake and to affect lipolysis in adipose tissue [31]. Other SNPs in *FTO* have been previously associated with BMI, obesity and type 2 diabetes [24]. Additionally, *FTO* was recently associated with HDL-C in a large, gene centric analysis [25], and it was suggested that this association with *FTO* and HDL-C may be mediated through *FTO*’ s association with BMI, since the observed association with *FTO* and HDL-C lost significance after BMI adjustment [25]. However, in our data, when we add BMI to the model, rs12595985 remains associated with HDL-C response to atenolol (*P*=3x10^-4^), suggesting the association may be through a different mechanism. Additionally, while we did not observe a significant difference in *FTO* expression by rs12595985 genotype at baseline, this does not preclude that expression differences might exist in more relevant tissues (e.g. liver).

Other top genes are also of note. *ABCB1*, also known as *MDR1* (multi-drug resistance protein 1), belongs to the MDR/TAP subfamily responsible for transporting various molecules across cell membranes. Evidence indicates a link of *ABCB1* variants with circulating lipid profiles, mainly LDL-C, and the efficacy of statins [26]. Furthermore, *ABCB1* 3435C>T (rs1045642) has been associated with efavirenz induced changes in HDL-C [32]. We did not observe association with HDL-C response to atenolol at this SNP in our study; however, because we did observe association in this gene, *ABCB1* and drug associated HDL-C change warrants further investigation. Next, *LRP5*, low-density lipoprotein receptor-related protein 5, encodes a transmembrane low-density lipoprotein receptor. Mice lacking the production of LPR5 had increased total plasma cholesterol levels and impaired glucose tolerance [27]. STARD3 N-terminal L, *STARD3NL*, encodes a late-endosomal protein that binds cholesterol and may assist in endosomal cholesterol transport [28], a process important in maintaining HDL cholesterol levels [33]. Also, *ESR1*, the estrogen receptor 1 gene, was shown associated with HDL-C response to atenolol. Variants in *ESR1* have been associated with many traits including type 2 diabetes in African Americans [34], and HDL-C response to atorvastatin in women [29]. The last gene of particular interest is *LIPC*, hepatic lipase, which is involved in lipoprotein metabolism, and other SNPs in the gene have also been associated with HDL-C in GWAS [11].

Many of the top gene regions identified have shown prior evidence of association with circulating HDL-C levels, HDL-C levels in response to drug therapy, or other metabolic traits or are involved in lipid metabolism. While this analysis was based on a candidate gene chip, the chip includes many genes involved in cardiovascular and inflammatory processes as well as genes involved in metabolic processes [12]. Thus, it is particularly interesting that a connection to HDL-C could be made for many of the gene regions meeting the consistent regional association requirement.

Like any study, the present investigation is not without limitations. For example, we had a relatively small sample size and did not have an external replication cohort. However, to our knowledge, there is not another suitable replication population with data on HDL-C response to a β-blocker at the present time. Also, due to our study design, we only focused on gene regions that showed a signal in both race groups. While true functional variants should be relevant to all race groups, it is very possible that we missed regions of interest due to our consistent regional association requirement and the differences in LD between the two race groups. Additionally, while we have identified gene regions of interest, we have likely not identified the functional variants in these regions. It is more likely we have identified SNPs in LD with the functional variant.

β-blockers are appropriate first-line therapy for hypertension [2]; however, there is growing concern regarding use in patients without compelling indications (i.e., heart failure or myocardial infarction), as the benefits associated with use of β-blockers may not outweigh the potential metabolic and other risks, including a decrease in HDL-C levels [3,4]. Our study investigated the possible genetic contributions to atenolol induced change in HDL-C in hypertensive study participants. We found 13 gene regions that showed initial evidence of association in one race (whites or African Americans), and consistent regional association in the other. Of particular interest were seven gene regions with prior associations with HDL-C or other metabolic traits, or functional implications in the lipid pathway: *GALNT2, FTO*, *ABCB1, LRP5*, *STARD3NL*, *ESR1*, and *LIPC*. We also observed differences in *GALNT2* baseline expression by rs2144300 genotype. These results require further investigation in order to confirm the associations, identify potential functional variants, elucidate their role, and establish the mechanism through which they act. However, our findings provide initial insight into genes that may influence atenolol induced change in HDL-C. 

## Supporting Information

Figure S1 **Manhattan Plots for the genome-spanning analysis with HDL-C response to atenolol in Whites (A) and African Americans (B).**
(TIFF)

Figure S2 **Q-Q plots for the genome-spanning analysis with HDL-C response to atenolol in Whites (A) and African Americans (B).**
(TIFF)

Table S1 **Allele frequencies, genotype counts, and Hardy-Weinberg Equilibrium P-values for the top signals and regional validation signals.**
(DOC)

Table S2 **Top gene regions associated with atenolol induced changes in HDL-C.** Data reflect top regions after a sensitivity analysis removing one White participant with an extreme HDL-C response. *P-value from sensitivity analysis after removing 1 White participant with an extreme HDL-C response. MA: Minor Allele; MAF: Minor Allele Frequency.
(XLSX)

Table S3 **Sensitivity analyses for *FTO* rs12595985 in African Americans for atenolol induced changes in HDL-C.** *Additive model after removing the 1 African American AA homozygote. Chr: Chromosome; MA: Minor Allele; MAF: Minor Allele Frequency.
(DOCX)

Table S4 **Predicted function of the top SNPs.** nsSNP: non-synonymous SNP.
(DOC)
