# Supplementary figures and images for: Atenolol Induced HDL-C Change in the Pharmacogenomic Evaluation of Antihypertensive Responses (PEAR) Study
Source: PLoS One. 2013 Oct 7;8(10):e76984. doi: 10.1371/journal.pone.0076984 (PMC3792156; doi:10.1371/journal.pone.0076984)

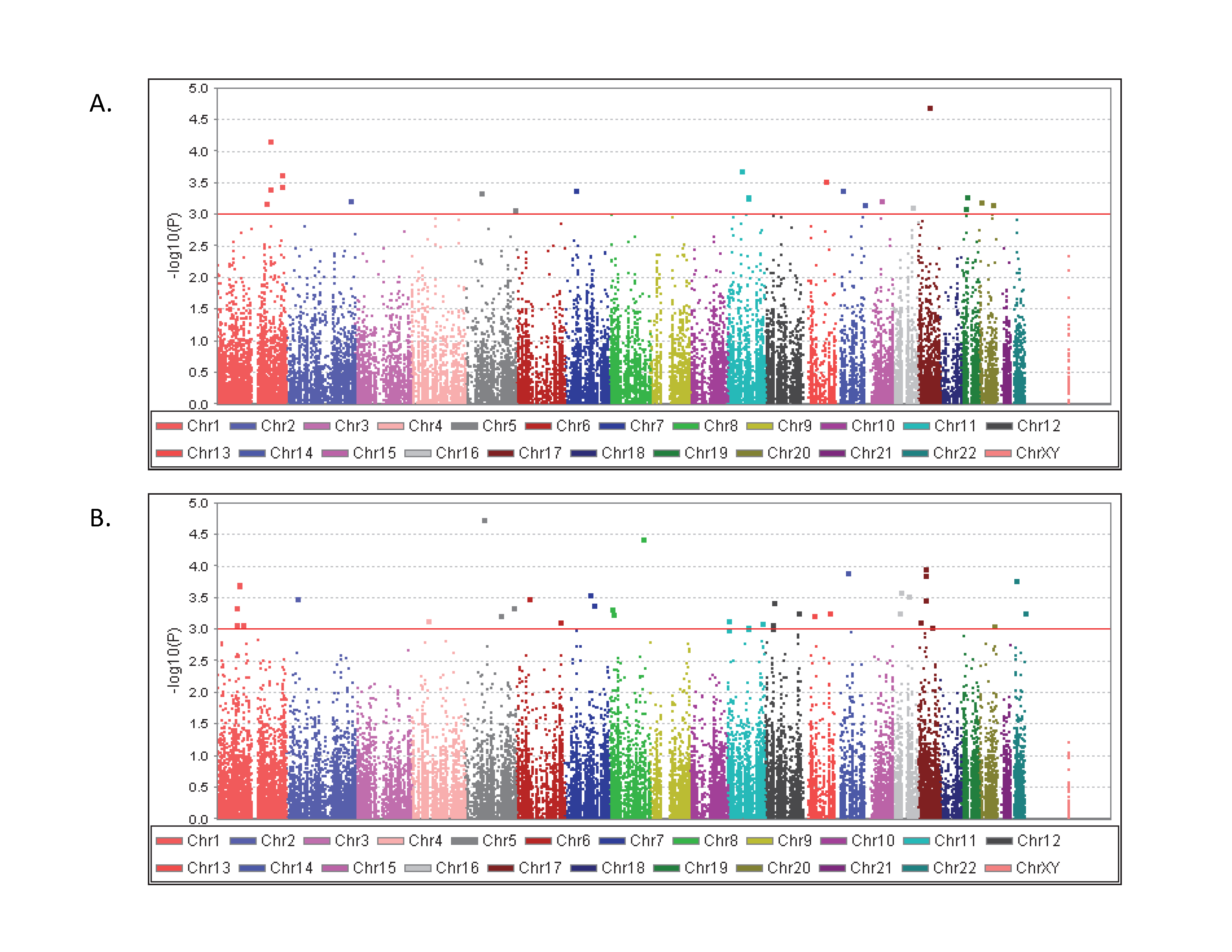

Supplement: Figure S1 — Manhattan Plots for the genome-spanning analysis with HDL-C response to atenolol in Whites (A) and African Americans (B). (TIFF) [file pone.0076984.s001.tiff]

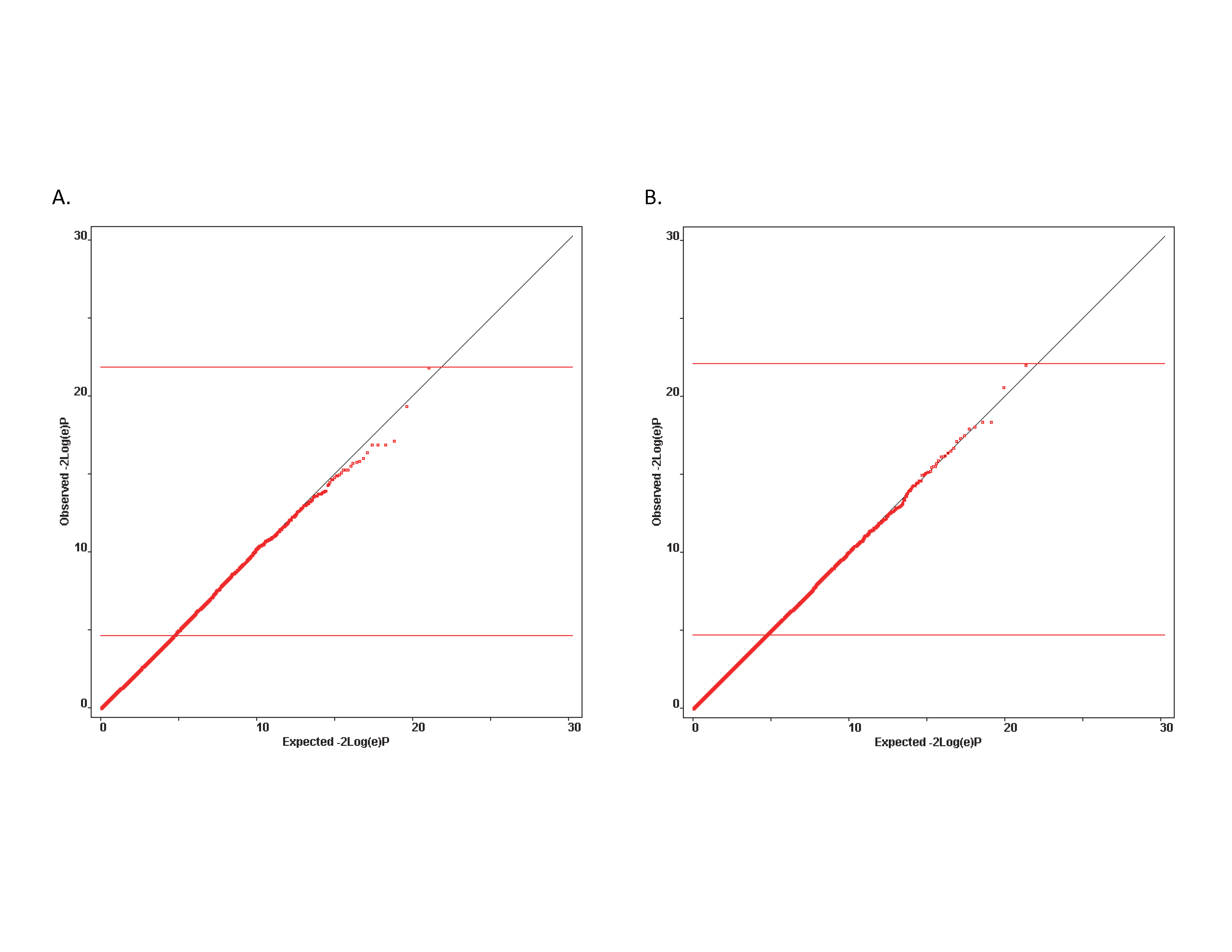

Supplement: Figure S2 — Q-Q plots for the genome-spanning analysis with HDL-C response to atenolol in Whites (A) and African Americans (B). (TIFF) [file pone.0076984.s002.tiff]
