# Supplementary material for: Atenolol Induced HDL-C Change in the Pharmacogenomic Evaluation of Antihypertensive Responses (PEAR) Study
Source: PLoS One. 2013 Oct 7;8(10):e76984. doi: 10.1371/journal.pone.0076984 (PMC3792156; doi:10.1371/journal.pone.0076984)
Supplement: Table S1 — Allele frequencies, genotype counts, and Hardy-Weinberg Equilibrium P-values for the top signals and regional validation signals. (DOC) [file pone.0076984.s003.doc]

**Table S1**.

| **SNP** | **Locus** | **Alleles** | | **Allele Frequencies** | | **Genotype Counts** | **Hardy-Weinberg  Equilibrium  P-value** |
| --- | --- | --- | --- | --- | --- | --- | --- |
|  |  | minor(m) | Major(M) | minor(m) | Major(M) | mm/mM/MM |  |
| **Whites - Initial Signals** | |  |  |  |  |  |  |
| **rs10157410** | PLA2G4A | C | G | 0.101 | 0.899 | 2/43/187 | 1 |
| **rs2144300** | GALNT2 | G | A | 0.422 | 0.578 | 49/98/85 | 0.044 |
| **rs10240718** | STARD3NL | A | G | 0.078 | 0.922 | 4/28/200 | 0.036 |
| **rs3736228** | LRP5 | A | G | 0.136 | 0.864 | 5/53/174 | 0.585 |
| **rs3818416** | EDNRB | A | C | 0.241 | 0.759 | 13/86/133 | 1 |
| **rs9652472** | LIPC | G | A | 0.041 | 0.959 | 2/15/215 | 0.047 |
| **rs3743725** | CDH16 | A | G | 0.030 | 0.970 | 0/14/218 | 1 |
| **African Americans - Initial Signals** | |  |  |  |  |  |  |
| **rs6847086** | REST | A | G | 0.461 | 0.540 | 30/80/42 | 0.517 |
| **rs3020384** | ESR1 | C | G | 0.487 | 0.513 | 34/80/38 | 0.626 |
| **rs3213619** | ABCB1 | G | A | 0.072 | 0.928 | 1/20/131 | 0.559 |
| **rs2975721** | MSRA | T | A | 0.385 | 0.615 | 22/73/57 | 1 |
| **rs7319001** | ABCC4 | A | G | 0.095 | 0.905 | 0/27/115 | 0.614 |
| **rs12595985** | FTO | A | C | 0.109 | 0.891 | 1/31/119 | 1 |
| **Whites - Regional Validation Signal** | | |  |  |  |  |  |
| **rs4109037** | REST | A | T | 0.136 | 0.864 | 4/55/173 | 1 |
| **rs12199198** | ESR1 | C | G | 0.067 | 0.933 | 3/25/203 | 0.069 |
| **rs10267099** | ABCA1 | G | A | 0.220 | 0.780 | 18/66/148 | 0.012 |
| **rs6601419** | MSRA | A | G | 0.338 | 0.662 | 26/105/101 | 1 |
| **rs1189470** | ABCC4 | C | A | 0.154 | 0.846 | 5/58/158 | 1 |
| **rs9940629** | FTO | A | G | 0.448 | 0.552 | 45/118/69 | 0.791 |
| **African Americans - Regional Validation Signal** | | |  |  |  |  |  |
| **rs4648287** | PLA2G4A | G | A | 0.145 | 0.855 | 2/40/110 | 0.741 |
| **rs2144297** | GALNT2 | A | G | 0.345 | 0.655 | 15/75/62 | 0.369 |
| **rs7795499** | STARD3NL | A | G | 0.329 | 0.671 | 15/70/67 | 0.714 |
| **rs4988331** | LRP5 | A | G | 0.168 | 0.832 | 7/37/108 | 0.140 |
| **rs3818416** | EDNRB | A | C | 0.329 | 0.671 | 22/56/74 | 0.044 |
| **rs10518978** | LIPC | A | G | 0.123 | 0.878 | 2/33/116 | 1 |
| **rs13336470** | CDH16 | A | G | 0.322 | 0.678 | 10/78/64 | 0.041 |
