# Supplementary material for: Atenolol Induced HDL-C Change in the Pharmacogenomic Evaluation of Antihypertensive Responses (PEAR) Study
Source: PLoS One. 2013 Oct 7;8(10):e76984. doi: 10.1371/journal.pone.0076984 (PMC3792156; doi:10.1371/journal.pone.0076984)
Supplement: Table S3 — Sensitivity analyses for FTO rs12595985 in African Americans for atenolol induced changes in HDL-C. *Additive model after removing the 1 African American AA homozygote. Chr: Chromosome; MA: Minor Allele; MAF: Minor Allele Frequency. (DOCX) [file pone.0076984.s005.docx]

**Table S3**.

| **Chr** | **Position (Mb)** | **Gene** | **Lead SNP** | **MA** | **MAF** | **Additive Model** | | **Dominant Model** | | **Additive Model*** | |
| --- | --- | --- | --- | --- | --- | --- | --- | --- | --- | --- | --- |
|  |  |  |  |  |  | Effect | *P*-value | Effect | *P*-value | Effect | *P*-value |
|  |  |  |  |  |  | (mg/dl) |  | (mg/dl) |  | (mg/dl) |  |
| 16 | 52.4 | FTO | rs12595985 | A | 0.109 | 4.52 | 2.90E-04 | 4.35 | 9.71E-04 | 3.90 | 3.18E-03 |
