# Supplementary material for: Atenolol Induced HDL-C Change in the Pharmacogenomic Evaluation of Antihypertensive Responses (PEAR) Study
Source: PLoS One. 2013 Oct 7;8(10):e76984. doi: 10.1371/journal.pone.0076984 (PMC3792156; doi:10.1371/journal.pone.0076984)
Supplement: Table S4 — Predicted function of the top SNPs. nsSNP: non-synonymous SNP. (DOC) [file pone.0076984.s006.doc]

**Table S4**.

| **Chr** | **Gene Region** | **SNP** | **Predicted function  (SNPNexus)** | **Predicted function  (FastSNP)** |
| --- | --- | --- | --- | --- |
| **Whites - Initial Signals** | | | | |
| **1** | PLA2G4A | rs10157410 | intronic | intronic |
| **1** | GALNT2 | rs2144300 | intronic | intronic |
| **7** | STARD3NL | rs10240718 | intronic | intronic |
| **11** | LRP5 | rs3736228 | nsSNP (A1330V) | nsSNP (A1330V);  splicing regulation |
| **13** | EDNRB | rs3818416 | intronic | intronic |
| **15** | LIPC | rs9652472 | intronic | intronic |
| **16** | CDH16 region | rs3743725 | downstream (3') | promoter/regulatory region |
| **African Americans - Initial Signals** | | | | |
| **4** | REST | rs6847086 | intronic | intronic enhancer |
| **6** | ESR1 | rs3020384 | intronic | intronic |
| **7** | ABCB1 | rs3213619 | intronic | intronic |
| **8** | MSRA | rs2975721 | intronic | intronic enhancer |
| **13** | ABCC4 | rs7319001 | intronic | intronic |
| **16** | FTO | rs12595985 | intronic | intronic enhancer |
